# Health-care workers’ occupational exposures to body fluids in 21 countries in Africa: systematic review and meta-analysis

**DOI:** 10.2471/BLT.17.195735

**Published:** 2017-10-13

**Authors:** Asa Auta, Emmanuel O Adewuyi, Amom Tor-Anyiin, David Aziz, Esther Ogbole, Brian O Ogbonna, Davies Adeloye

**Affiliations:** a School of Pharmacy and Biomedical Sciences, University of Central Lancashire, Fylde Road, Preston, PR1 2HE, England.; b Institute of Health and Biomedical Innovation, Queensland University of Technology, Brisbane, Australia.; c School of Health Sciences, Walden University, Minneapolis, United States of America.; d Biochemistry and Chemotherapy Division, Nigerian Institute for Trypanosomiasis Research, Vom, Nigeria.; e Faculty of Pharmaceutical Sciences, Nnamdi Azikiwe University, Awka, Nigeria.; f Department of Demography and Social Statistics, Covenant University, Ota, Nigeria.

## Abstract

**Objective:**

To estimate the lifetime and 12-month prevalence of occupational exposure to body fluids among health-care workers in Africa.

**Methods:**

Embase®, PubMed® and CINAHL databases were systematically searched for studies published between January 2000 and August 2017 that reported the prevalence of occupational exposure to blood or other body fluids among health-care workers in Africa. The continent-wide prevalence of exposure was estimated using random-effects meta-analysis.

**Findings:**

Of the 904 articles identified, 65 studies from 21 African countries were included. The estimated pooled lifetime and 12-month prevalence of occupational exposure to body fluids were 65.7% (95% confidence interval, CI: 59.7–71.6) and 48.0% (95% CI: 40.7–55.3), respectively. Exposure was largely due to percutaneous injury, which had an estimated 12-month prevalence of 36.0% (95% CI: 31.2–40.8). The pooled 12-month prevalence of occupational exposure among medical doctors (excluding surgeons), nurses (including midwives and nursing assistants) and laboratory staff (including laboratory technicians) was 46.6% (95% CI: 33.5–59.7), 44.6% (95% CI: 34.1–55.0) and 34.3% (95% CI: 21.8–46.7), respectively. The risk of exposure was higher among health-care workers with no training on infection prevention and those who worked more than 40 hours per week.

**Conclusion:**

The evidence available suggests that almost one half of health-care workers in Africa were occupationally exposed to body fluids annually. However, a lack of data from some countries was a major limitation. National governments and health-care institutions across Africa should prioritize efforts to minimize occupational exposure among health-care workers.

## Résumé

**Objectif:**

Estimer la prévalence au cours de la vie et sur 12 mois de l'exposition professionnelle aux liquides organiques des agents de santé en Afrique.

**Méthodes:**

Nous avons systématiquement recherché dans les bases de données Embase®, PubMed® et CINAHL des études publiées entre janvier 2000 et août 2017 documentant la prévalence de l'exposition professionnelle au sang ou à d'autres liquides organiques des agents de santé en Afrique. La prévalence de l'exposition dans l'ensemble du continent a été estimée à l'aide d'une méta-analyse à effets aléatoires.

**Résultats:**

Sur les 904 articles repérés, 65 études menées dans 21 pays africains ont été sélectionnées. La prévalence combinée au cours de la vie et sur 12 mois de l'exposition professionnelle aux liquides organiques était estimée à 65,7% (intervalle de confiance, IC à 95%: 59,7–71,6) dans le premier cas et 48,0% (IC à 95%: 40,7–55,3) dans le second. L'exposition était en grande partie due à des lésions percutanées, la prévalence sur 12 mois étant estimée à 36,0% (IC à 95%: 31,2-40,8). La prévalence combinée sur 12 mois de l'exposition professionnelle était de 46,6% (IC à 95%: 33,5–59,7) chez les médecins (à l'exception des chirurgiens), de 44,6% (IC à 95%: 34,1–55,0) chez les infirmiers (sages-femmes et infirmiers auxiliaires compris) et de 34,3% (IC à 95%: 21,8–46,7) chez le personnel de laboratoire (techniciens de laboratoire compris). Le risque d'exposition était plus élevé chez les agents de santé n'ayant pas été formés à la prévention des infections et chez ceux qui travaillaient plus de 40 heures par semaine.

**Conclusion:**

D'après les données disponibles, près de la moitié des agents de santé en Afrique sont exposés chaque année aux liquides organiques dans le cadre de leur travail. Le manque de données dans certains pays a néanmoins constitué une limite majeure. Les gouvernements nationaux et les établissements de santé de l'ensemble du continent doivent donner un degré de priorité élevé aux efforts visant à minimiser l'exposition professionnelle des agents de santé.

## Resumen

**Objetivo:**

Hacer una estimación del tiempo de vida y la prevalencia de 12 meses de exposición profesional a fluidos corporales entre los profesionales sanitarios en África.

**Métodos:**

Se realizó una búsqueda sistemática en las bases de datos Embase®, PubMed® y CINAHL de estudios publicados entre enero de 2000 y agosto de 2017 que mostraran la prevalencia de la exposición profesional a sangre u otros fluidos corporales entre los profesionales sanitarios en África. La prevalencia de la exposición en todo el continente se estimó utilizando un meta-análisis de efectos aleatorios.

**Resultados:**

De los 904 artículos identificados se incluyeron 65 estudios de 21 países africanos. El tiempo de vida estimado en conjunto y la prevalencia de 12 meses de exposición profesional a fluidos corporales fue de un 65,7% (intervalo de confianza, IC, 95%: 59,7–71,6) y 48,0% (95% IC: 40,7–55,3), respectivamente. La exposición se debía en su mayor parte a heridas percutáneas, con una prevalencia estimada durante 12 meses del 36,0% (95% IC: 31,2–40,8). La prevalencia durante 12 meses de exposición profesional en conjunto entre los médicos (excepto los cirujanos), enfermeras (incluidas las matronas y las auxiliares de enfermería) y el personal de laboratorio (incluidos los técnicos de laboratorio) fue del 46,6% (95% IC: 33,5–59,7), 44,6% (95% IC: 34,1–55,0) y del 34,3% (95% IC: 21,8-46,7), respectivamente. El riesgo de exposición fue más alto entre los profesionales sanitarios sin formación en el ámbito de la prevención de infecciones y entre aquellos que trabajaban más de 40 horas a la semana.

**Conclusión:**

Las pruebas disponibles sugieren que casi la mitad de todos los trabajadores sanitarios en África están expuestos profesionalmente a fluidos corporales cada año. Sin embargo, la falta de datos de algunos países supuso una gran limitación. Por lo tanto, los gobiernos nacionales y las instituciones sanitarias africanas deberían priorizar los esfuerzos para disminuir la exposición entre los profesionales sanitarios.

## ملخص

**الغرض:**

تقييم انتشار التعرض لملامسة سوائل الجسم بين العاملين في مجال الرعاية الصحية في أفريقيا في سياق الممارسات المهنية، وذلك على مدار 12 شهرًا وطوال الحياة.

**الطريقة:**

جرى البحث المنهجي في قواعد البيانات Embase® وPubMed® وCINAHL للوصول إلى الدراسات التي سبق نشرها في الفترة بين يناير/كانون الثاني 2000 وأغسطس/آب 2017 والتي تسجل انتشار التعرض لملامسة الدم أو غيره من سوائل الجسم بين العاملين في مجال الرعاية الصحية في أفريقيا في سياق الممارسات المهنية. وتم تقييم انتشار تلك الحالات على نطاق القارة بأكملها باستخدام تحليل تلوي للآثار العشوائية.

**النتائج:**

قمنا بتضمين 65 دراسة من 21 دولة أفريقية من بين 904 مقالات تم تحديدها. بلغت نسبة الحالات المجمعة لانتشار التعرض لسوائل الجسم في سياق الممارسات المهنية على مدى 12 شهرًا أو مدى الحياة وفقًا للتقديرات ‏65.7% (بنطاق ثقة تبلغ نسبته: 59.7 – 71.6) و48.0‏% (بنطاق ثقة تبلغ نسبته ‏95%: 40.7 - 55.3)، على التوالي. وكان التعرض لملامسة تلك السوائل يرجع في الأغلب إلى الإصابات عن طريق الجلد، والتي بلغت نسبة انتشارها على مدى 12 شهرًا ‏36.0% (بنطاق ثقة تبلغ نسبته 95‏%: 31.2–40.8). وبلغت النسبة المجمعة لحالات انتشار التعرض لملامسة تلك السوائل في سياق الممارسات المهنية على مدى 12 شهرًا لدى الأطباء المعالجين (باستثناء الجراحين)، والممرضين (بما يشمل القابلات ومساعدي الممرضين) وفريق العمل بالمعامل (بما يشمل فنيي المعامل) 46.6% (بنطاق ثقة تبلغ نسبته 95‏%: 33.5 – 59.7)، و44.6‏% (بنطاق ثقة تبلغ نسبته 95‏%: 34.1 – 55.0) و34.3‏% (بنطاق ثقة تبلغ نسبته ‏95%: 21.8 – 46.7)، على التوالي. وارتفع خطر التعرض لملامسة تلك السوائل بين العاملين في مجال الرعاية الصحية الذين لم يتلقوا تدريبًا على الوقاية من العدوى ومن كان منهم يعمل لمدة تزيد عن 40 ساعة في الأسبوع.

**الاستنتاج:**

أشارت الأدلة المتاحة إلى أن ما يقرب من نصف العاملين في مجال الرعاية الصحية في أفريقيا يتعرضون سنويًا لملامسة سوائل الجسم في سياق الممارسات المهنية. وبالرغم من ذلك، كان النقص في البيانات المتعلقة ببعض البلدان عائقًا رئيسيًا. ويجب على الحكومات الوطنية ومؤسسات الرعاية الصحية المنتشرة في أنحاء أفريقيا أن تضع ضمن أولوياتها الجهود الرامية للتقليل إلى أدنى حد من تعرض العاملين في مجال الرعاية الصحية لتلك الحالات في إطار الممارسات المهنية.

## 摘要

**目的:**

旨在估算非洲医疗护理工作者在工作过程中接触体液的终生和年患病率。

**方法:**

系统地搜索 Embase®、PubMed® 和 CINAHL 数据库中在 2000 年 1 月至 2017 年 8 月间发表的报告非洲医疗护理工作者在工作过程中接触血液或其他体液的研究。采用随机效应元分析估算非洲大陆范围内由于接触而感染的患病率。

**结果:**

在选定的 904 篇文章中，收录来自 21 个国家的 65 份研究。由于在工作中接触体液而感染的终身和年患病率总估计值分别为 65.7%（95% 置信区间，CI: 59.7–71.6）和 48.0% (95% CI: 40.7–55.3)。接触大部分是由于经皮损伤，年患病率汇总估计值为 36.0% (95% CI: 31.2–40.8)。医生（不包括外科医生）、护士（包括助产士和护理员）和实验室人员（包括实验室技术人员）在工作过程中由于接触而感染的汇总年患病率分别为 46.6% (95% CI: 33.5–59.7)、44.6% (95% CI: 34.1–55.0) 和 34.3% (95% CI: 21.8–46.7)。未经感染预防培训和每周工作时间超过 40 小时的医疗护理工作者接触的风险偏高。

**结论:**

现有的证据表明每年约有近半数的非洲医疗护理工作者会在工作过程中接触体液。然而，缺乏一些国家的数据是主要局限因素。非洲各国政府和医疗护理机构应优先考虑将医疗护理工作者在工作过程中接触体液的风险降到最低。****

## Резюме

**Цель:**

Оценить распространенность случаев профессионального вредного воздействия биологических жидкостей среди медицинских работников в Африке на протяжении жизни и за 12 месяцев.

**Методы:**

Был проведен систематический поиск в базах данных Embase®, PubMed® и CINAHL на предмет исследований, опубликованных в период с января 2000 года по август 2017 года, в которых сообщалось о распространенности случаев профессионального вредного воздействия крови или других биологических жидкостей среди медицинских работников в Африке. Общая распространенность вредного воздействия на континенте оценивалась с помощью метаанализа с использованием модели случайных эффектов.

**Результаты:**

Из 904 выявленных статей в отчет было включено 65 исследований из 21 африканской страны. Предполагаемая распространенность случаев профессионального вредного воздействия биологических жидкостей в течение обобщенной продолжительности жизни и за 12 месяцев составили 65,7% (95%-ный доверительный интервал, ДИ: 59,7–71,6) и 48,0% (95%-ный ДИ: 40,7–55,3) соответственно. Вредное воздействие было в большинстве случаев обусловлено чрескожной травмой, 12-месячная распространенность которой, по оценкам, составила 36,0% (95%-ный ДИ: 31,2–40,8). Суммарная 12-месячная распространенность случаев профессионального вредного воздействия среди врачей (исключая хирургов), медсестер (включая акушерок и помощников медсестер) и сотрудников лаборатории (включая лаборантов) составила 46,6% (95%-ный ДИ: 33,5–59,7), 44,6% (95%-ный ДИ: 34,1–55,0) и 34,3% (95%-ный ДИ: 21,8–46,7) соответственно. Риск вредного воздействия был выше среди медицинских работников, не прошедших обучение по профилактике инфекции, и среди тех, кто работал более 40 часов в неделю.

**Вывод:**

Имеющиеся данные свидетельствуют о том, что в Африке около половины медицинских работников ежегодно подвергаются вредному воздействию биологических жидкостей. Однако отсутствие данных из некоторых стран являлось основным ограничением. Национальным правительствам и учреждениям здравоохранения в Африке следует уделять первоочередное внимание усилиям по минимизации риска профессионального вредного воздействия среди работников здравоохранения.

## Introduction

Worldwide, health-care workers risk occupational exposure to blood-borne pathogens through contact with human body fluids. Although about 60 blood-borne infectious pathogens have been identified, including Epstein–Barr virus, most occupation-related, blood-borne infections are due to hepatitis B virus (HBV), hepatitis C virus (HCV) and human immunodeficiency virus (HIV).^1^^,^^2^ However, other blood-borne pathogens still pose a risk: for example, in the 2013–2016 Ebola virus disease outbreak, over 890 health-care workers were infected, with a case fatality rate of 57%.^3^ Occupational exposure can occur through percutaneous injury (i.e. a needle or sharp object penetrates the skin), mucous membrane exposure (e.g. of the eyes, nose or mouth) and non-intact skin exposure. Percutaneous injury accounts for 66 to 95% of occupational exposures to blood-borne pathogens.^4^

Little is known about the global burden of percutaneous injury among health-care workers. However, a 2005 report estimated that worldwide more than 3 million occupation-related percutaneous injuries occur annually.^4^ Moreover, about 40% of HBV and HCV infections and 2.5% of HIV infections in health-care workers were due to percutaneous injuries.^5^ Hence, each year, percutaneous injury resulted in around 66 000 HBV infections, 16 000 HCV infections and 1000 HIV infections, which together caused about 1100 deaths as well as substantial disability.^4^ More than 90% of these infections occurred in developing countries, particularly in Africa, where infection is more prevalent and adherence to standard precautions can be poor.^5^

Given the severe consequences of blood-borne infections, many high-income countries have established surveillance systems to monitor exposure to body fluids in health-care settings.^6^ These systems help inform policy-makers for reducing the risk of transmission of blood-borne pathogens. In many African countries, such systems are not available and, consequently, exposure to body fluids is rarely monitored. Furthermore, occupational exposure of health-care workers in Africa is generally underreported and poorly documented – one Nigerian study found that up to 97% of exposures were not reported.^7^

The true incidence of blood and body fluid exposure in Africa is, therefore, uncertain. The 2005 report estimated that the incidence of sharps injuries in individual health-care workers in Africa was 2.10 per annum.^4^ However, the authors based the estimate on survey findings from eight African countries and did not include data on laboratory technicians or other auxiliary health-care workers. Moreover, the authors obtained the data in hospitals and may not be representative of the diverse range of health-care settings in the continent. A Congolese study found an annual prevalence of occupational exposure to body fluids among health-care workers of 44.9%, with an average of 1.38 exposures per health-care worker per year.^8^ A Burundian study reported an annual prevalence of 67.6%, with an average of 2.7 exposures per health-care worker per year.^9^

Here we conducted a systematic review of observational studies to estimate the prevalence of occupational exposure to blood and body fluids among health-care workers in Africa, because a continent-wide estimate would help increase awareness of such exposure and prompt preventative measures.

## Methods

We searched the Embase®, CINAHL and PubMed® databases on 1 September 2017 for original research articles published between January 2000 and August 2017 that reported the prevalence of occupational exposure to blood or other body fluids among health-care workers in Africa. The following search terms were combined with others using Boolean operators: “occupational exposure”, “accidental exposure”, “blood”, “body fluid”, “blood-borne pathogens”, “health-care workers”, “health workers”, “health personnel” and “Africa*”* (Box 1; available at: http://www.who.int/bulletin/volumes/95/12/17-195735). Additional articles were identified by checking reference lists and by Google and Google Scholar searches. There were no language restrictions. The research protocol was registered in the PROSPERO international prospective register of systematic reviews (CRD42017054288).

Box 1 **Search strategy, systematic review, blood and body fluid exposure among health-care workers in Africa, 2000–2017**

1. (Occupation* exposure OR Accident* exposure OR Occupation* disease OR Accidental blood disease* OR Accidental occupational exposure OR Occupational hazard* OR Occupational transmission OR Cross infection).af.
2. (Blood OR Body fluid* OR blood spill* OR needle injur* OR Blood borne pathogen* OR Sharps* OR Needlestick injur* OR Needle stick OR Blood-borne infection* OR percutaneous injur* OR mucus membrane exposure* OR non-intact skin exposure* OR bite* OR cut* OR Human immunodeficiency virus OR HIV OR Hepatitis B OR Hepatitis C).af.
3. (Health care worker* OR Nurse* OR Midwive* OR Physician* OR Surgeon* OR Doctor* OR Health personnel OR Health worker* OR Dentist* OR Health staff OR Medical personnel OR Health personnel OR Health officer*).af.
4. (Africa OR Nigeria OR Senegal OR Morocco OR South Africa OR Ethiopia OR Kenya OR Mauritius OR Mauritania OR Tanzania OR Congo OR Algeria OR Tunisia OR Libya OR Ghana OR Madagascar OR Gabon OR Cameroon OR Mali OR Zimbabwe OR Sudan OR Uganda OR Somalia OR Namibia OR Angola OR Mozambique OR Rwanda OR Eritrea OR Burkina Faso OR Gambia OR Zambia OR Botswana OR Guinea OR Djibouti OR Niger OR Malawi OR Togo OR Liberia OR Benin OR Sierra Leone OR Swaziland OR Côte d’Ivoire OR Chad OR Seychelles OR Cape Verde OR Burundi OR Lesotho).af.
5. 1 AND 2 AND 3 AND 4
6. Limit 5 to yr = ”2000–Current”

For this review, we considered occupational exposure to body fluids to occur through percutaneous injury, mucous membrane exposure, non-intact skin exposure and bites. We included studies that reported the lifetime or 12-month prevalence of occupational exposure through at least one of these routes. Health-care workers included all paid and unpaid individuals working in a health-care setting who could be exposed to infectious materials, including blood and body fluids. Hence, we included studies that involved doctors, nurses, laboratory technicians, auxiliary health-care workers or students undertaking clinical training or gaining experience in health-care settings. In addition, we included studies if they were observational studies with either a cohort or cross-sectional design. We excluded case reports, case series, case–control studies, qualitative studies, studies with fewer than 100 participants and, because of historic underreporting in Africa, studies that reviewed reported cases of blood and body fluid exposure. Two reviewers independently screened studies against inclusion and exclusion criteria (kappa for inter-rater agreement: 90.8%). Discrepancies were resolved by consensus.

The quality of each study was assessed and the risk of bias was judged using eight parameters, modelled largely on the Joanna Briggs Institute’s critical appraisal framework for prevalence studies: the sampling frame, sample size, sampling strategy, detailed description of research setting and population, response rate (adequate if 60% or higher), reliability of the instrument used, recall bias (12 months or shorter) and statistical analysis methods – failure to satisfy each parameter was scored as 1.^10^ The risk of bias was classified as either low (total score: 0 to 2), moderate (total score: 3 or 4) or high (total score: 5 to 8).

Two reviewers extracted data from the studies and entered them into Microsoft Excel v. 16.0 (Microsoft Corporation, Redmond, United States of America). The data included: (i) author; (ii) year of publication; (iii) study country; (iv) sample size; (v) response rate; (vi) recall period; (vii) prevalence of blood and body fluid exposure; (viii) prevalence of percutaneous injury; (ix) prevalence of mucous membrane and non-intact skin exposure; (x) prevalence of blood and body fluid exposure by health staff category; and (xi) the proportions of cases due to needle-stick injury, splashes, cuts and bites. Any discrepancy was resolved by consensus.

Two countries, Egypt and Libya, are included in WHO’s Eastern Mediterranean Region, but were classified as African for the purposes of this analysis.

### Data analysis

We categorized studies by whether they measured lifetime or 12-month prevalence and by the type of blood and body fluid exposure considered: (i) all types, including percutaneous injury and mucous membrane exposure; or (ii) percutaneous injury only. Generally, we estimated lifetime prevalence using data from studies that reported the proportion of participants exposed to body fluids at any time during their career. Twelve-month prevalence was estimated using data from studies that reported the proportion of participants exposed to body fluids in the preceding 12 months. We derived pooled prevalence estimates of blood and body fluid exposure by random-effects meta-analysis based on the DerSimonian–Laird approach.^11^ We assessed the robustness of our findings in sensitivity analyses that excluded studies with a high risk of bias.

Interstudy heterogeneity was assessed by Cochran’s Q, which gives values for *X^2^* and *P*, and the percentage of the total variation across studies due to heterogeneity was estimated using Higgin’s *I^2^* statistic.^12^ The causes of heterogeneity were explored in subgroup and meta-regression analyses. We considered the covariates: (i) geographical region; (ii) type of health-care facility; (iii) study period; (iv) sampling procedure (i.e. random versus convenience sampling); (v) sample size; (vi) proportion of doctors; (vii) proportion of nurses; (viii) proportion of laboratory staff; and (ix) the risk of bias classification. Only those covariates found to be significant at *P* < 0.10 were included in the multivariate model. In addition, the pooled prevalence of blood and body fluid exposure in different categories of health-care worker were derived in stratified analyses and the relative risk of occupational exposure between groups was determined by pooling data using a random-effects model. We performed all statistical analyses using Stata version 13.1 (StataCorp LP., College Station, USA).

## Results

**Fig. 1:**
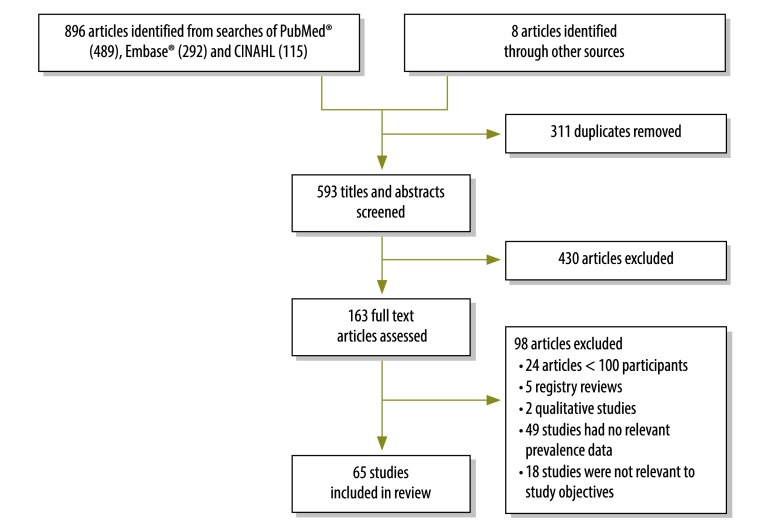
Flow diagram, systematic review, blood and body fluid exposure among health-care workers in Africa, 2000–2017

We identified 904 articles through the literature search, of which 65 were eligible for inclusion: they reported on cross-sectional observational studies involving a total of 29 385 health-care workers from 21 African countries (Fig. 1).^7^^–^^9^^,^^13^^–^^74^ Of the 65 studies, 30 were conducted in eastern Africa, 18 in western Africa, eight in northern Africa, five in southern Africa and four in central Africa (Table 1; available at: http://www.who.int/bulletin/volumes/95/12/17-195735). Thirty-nine studies were done solely among hospital staff, 39 investigated blood and body fluid exposure through all routes and 26 investigated exposure through percutaneous injury only. We found low risk of bias in 37 studies, moderate risk in 25 and high risk in 3; in 44 studies, the increased risk of bias was largely due to sampling bias.

**Table 1:** Studies identified in the systematic review on blood and body fluid exposure among health-care workers in Africa, 2000–2017

Study authors and year	Country and continental region	Data reported	Study participants and setting^a^	Prevalence of all types of exposure to BBF, %	Prevalence of PCI, %	Risk of bias^b^
Newsom and Kiwanuka,^13^ 2002	Uganda, eastern Africa	12-month prevalence of PCI	180 doctors, nurses and laboratory staff in Mbarara Teaching Hospital	N/A	12-month: 55.0	Low
Le Pont et al.,^9^ 2003	Burundi, central Africa	Lifetime and 12-month prevalence of all types of exposure to BBF and disaggregated PCI data	219 doctors, nurses, nursing assistants and auxiliary staff in Kamenge University Hospital, Bujumbura	Lifetime: 79.5; 12-month: 67.6	12-month: 55.0	Low
Talaat et al.,^14^ 2003	Egypt, northern Africa	Lifetime prevalence of PCI	1845 doctors, dentists, nurses and laboratory and auxiliary staff in 98 health-care facilities (i.e. government hospitals, primary care facilities and private facilities) in two Governorates (Nile Delta and Upper Egypt)	N/A	Lifetime: 69.4	Moderate
Bodkin and Bruce,^15^ 2003	South Africa, southern Africa	12-month prevalence of PCI	159 doctors, nurses and medical and nursing students in a teaching hospital in Gauteng	N/A	12-month: 16.4	Low
Tarantola et al.,^16^ 2005	Côte d'Ivoire, Mali and Senegal, western Africa	12-month prevalence of all types of exposure to BBF and disaggregated data on PCI	1241 doctors, nurses, laboratory staff and clinical students in 43 hospital departments and transfusion clinics in Abidjan (Côte d’Ivoire), Bamako (Mali) and Dakar (Senegal)	12-month: 45.7	12-month: 38.1	Moderate
Ismail et al.,^17^ 2005	Egypt, northern Africa	Lifetime prevalence of PCI	1100 doctors and nurses in 7 hospitals and 18 primary health-care centres in Gharbiya Governorate	N/A	Lifetime: 66.2	Moderate
Obi et al.,^18^ 2005	Nigeria, western Africa	Lifetime prevalence of PCI	264 surgeons in five tertiary health institutions in south-eastern Nigeria	N/A	Lifetime: 66.7	Moderate
Nsubuga and Jaakkola,^19^ 2005	Uganda, eastern Africa	Lifetime and 12-month prevalence of PCI	526 nurses and midwives in Mulago national referral hospital in Kampala, Uganda	N/A	Lifetime: 82; 12-month: 57	Low
National AIDS and STD Control Programme,^20^ 2006	Kenya, eastern Africa	12-month prevalence of all types of exposure to BBF	1897 doctors, clinical officers, nurses, laboratory technicians, social workers and other support staff across a nationally representative sample of 247 health-care facilities	12-month: 17.0	ND	Low
Ibekwe and Ibeziako,^7^ 2006	Nigeria, western Africa	Lifetime prevalence of PCI	246 doctors, nurses, laboratory technicians and ward attendants in University of Nigeria Teaching Hospital, Enugu	N/A	Lifetime: 53.7	High
Braka et al.,^21^ 2006	Uganda, eastern Africa	Lifetime prevalence of PCI	311 doctors, dental staff, nurses, laboratory staff, midwives and auxiliary staff in 48 districts in Uganda	N/A	Lifetime: 77.2	Low
Kabbash et al.,^22^ 2007	Egypt, northern Africa	12-month prevalence of PCI	317 doctors and nurses from 32 haemodialysis units in the Nile delta	N/A	12-month: 48.6	Low
Sofola et al.,^23^ 2007	Nigeria, western Africa	Lifetime prevalence of all types of exposure to BBF	153 clinical dental students in four dental training institutions in Lagos, Ibadan, Ife and Benin	Lifetime: 58.8	ND	Moderate
De Villiers et al.,^24^ 2007	South Africa, southern Africa	Lifetime prevalence of all types of exposure to BBF	228 doctors in public and private practice in Bloemfontein	Lifetime: 54.2	ND	Low
Taegtmeyer et al.,^25^ 2008	Kenya, eastern Africa	12-month prevalence of PCI	554 doctors, nurses and counsellors in 11 health facilities: two hospitals, eight health centres and one dispensary, Thika District	N/A	12-month: 30	Low
Laraqui et al.,^26^ 2008	Morocco, northern Africa	Lifetime and 12-month prevalence of all types of exposure to BBF	2086 doctors, nurses and laboratory and support staff in 10 hospitals in 10 cities	Lifetime: 76.6; 12-month: 58.9	ND	Low
Okeke et al.,^27^ 2008	Nigeria, western Africa	Lifetime prevalence of PCI	346 medical students in a university	N/A	Lifetime:48	Moderate
Manyele et al.,^28^ 2008	United Republic of Tanzania, eastern Africa	Lifetime prevalence of all types of exposure to BBF and disaggregated data on PCI	430 nurses and attendants in 14 district, regional and referral hospitals	Lifetime: 74.6	Lifetime: 52.9	Moderate
Laraqui et al.,^29^ 2009	Morocco, northern Africa	Lifetime and 12-month prevalence of all types of exposure to BBF	1002 doctors, nurses and support staff in four hospitals in the cities of Meknes, Taza, Tiznit and Rabat	Lifetime: 89.2; 12-month: 62.8	ND	Low
Reda et al.,^30^ 2010	Ethiopia, eastern Africa	Lifetime and 12-month prevalence of all types of exposure to BBF and disaggregated data on PCI	484 doctors, nurses, midwives, laboratory technicians, health officers and assistants in 10 hospitals and 20 health centres, eastern Ethiopia	Lifetime: 85.0; 12-month: 51.2	Lifetime: 56.2; 12-month: 31.0	Low
Tadesse and Tadesse,^31^ 2010	Ethiopia, eastern Africa	Lifetime and 12-month prevalence of PCI	366 nurses and laboratory technicians in 26 health facilities including a university teaching hospital and one private hospital in Awassa City, southern Ethiopia	N/A	Lifetime: 49.2; 12-month: 30.9	Low
Tebeje and Hailu,^32^ 2010	Ethiopia, eastern Africa	Lifetime prevalence of all types of exposure to BBF and disaggregated data on PCI	254 doctors, nurses, midwives, laboratory technicians and health officers in government health facilities in Jimma zone and Jimma City	Lifetime: 68.5	Lifetime: 41.3	Moderate
Azodo,^33^ 2010	Nigeria, western Africa	Lifetime prevalence of PCI	300 dentists across Nigeria	N/A	Lifetime: 69.3	High
Hanafi et al.,^34^ 2011	Egypt, northern Africa	12-month prevalence of PCI	645 doctors, nurses and auxiliary staff in University of Alexandria teaching hospitals	N/A	12-month: 67.9	Low
Nwankwo and Aniebue,^35^ 2011	Nigeria, western Africa	12-month prevalence of all types of exposure to BBF	184 trainee surgeons in three hospitals in Enugu, south-eastern Nigeria	12-month: 67.5	ND	Moderate
Elduma and Saeed,^36^ 2011	Sudan, eastern Africa	Lifetime prevalence of PCI	245 doctors, dentists, nurses and laboratory and support staff in three teaching hospitals, Khartoum	N/A	Lifetime: 51	Moderate
Kumakech et al.,^37^ 2011	Uganda, eastern Africa	12-month prevalence of all types of exposure to BBF and disaggregated data on PCI	224 doctors, nurses, midwives, laboratory staff and medical and nursing students in Mbarara Regional Referral Hospital, south-western Uganda	12-month: 33.9	12-month: 23.6	Low
Ngatu et al.,^8^ 2012	Democratic Republic of the Congo, central Africa	12-month prevalence of all types exposure to BBF	1043 doctors, nurses and laboratory and support staff in four urban and rural hospitals in the southern town of Lubumbashi and the western semirural city of Matadi	12-month: 44.9	ND	Moderate
Shiferaw et al.,^38^ 2012	Ethiopia, eastern Africa	12-month prevalence of all types of exposure to BBF and disaggregated data on PCI	126 medical waste handlers in three government hospitals in Addis Ababa	12-month: 67.5	12-month: 42.1	Moderate
Pellissier et al.,^39^ 2012	Niger, western Africa	Lifetime prevalence of PCI	207 nurses and medical, paramedical, cleaning and administrative staff in Niamey’s National Hospital	N/A	Lifetime: 40.1	Moderate
Owolabi et al.,^40^ 2012	Nigeria, western Africa	12-month prevalence of all types of exposure to BBF and disaggregated data on PCI	230 doctors, nurses and laboratory staff in University of Abuja Teaching Hospital	12-month: 30.9	12-month: 24.8	Low
Odongkara et al.,^41^ 2012	Uganda, eastern Africa	Lifetime prevalence of all types of exposure to BBF	235 doctors, nurses and laboratory staff in Gulu Regional Referral Hospital and St. Mary's Hospital Lacor, northern Uganda	Lifetime: 46	ND	Moderate
Noubiap et al.,^42^ 2013	Cameroon, central Africa	Lifetime prevalence of all types of exposure to BBF	111 clinical medical students of the Faculty of Medicine and Biomedical Sciences of the University of Yaoundé	Lifetime: 55.9	ND	Moderate
Zawilla and Ahmed,^43^ 2013	Egypt, northern Africa	12-month prevalence of PCI	1036 health-care workers in Cairo University Hospitals	N/A	12-month: 40	Low
Mathewos et al.,^44^ 2013	Ethiopia, eastern Africa	Lifetime prevalence of all types of exposure to BBF	195 doctors, nurses, laboratory technicians, midwives, anaesthetists, heath officers and physiotherapists in Gondar University Hospital	Lifetime: 33.8	ND	Low
Yimechew and Tadese Ejigu,^45^ 2013	Ethiopia, eastern Africa	Lifetime and 12-month prevalence of all types of exposure to BBF and disaggregated data on PCI	285 doctors, nurses, laboratory staff, auxiliary staff and medical students in the University of Gondar Hospital	Lifetime: 70.2; 12-month: 62.9	12-month: 41	Low
Mbaisi et al.,^46^ 2013	Kenya, eastern Africa	12-month prevalence of all types of exposure to BBF and disaggregated data on PCI	305 doctors, clinical officers, nurses, laboratory personnel, mortuary attendants, housekeeping staff and clinical students in Rift Valley Provincial General Hospital	12-month: 25	12-month: 19	Low
Osazuwa-Peters et al.,^47^ 2013	Nigeria, western Africa	12-month prevalence of PCI	144 medical and dental house officers in three government hospitals in Edo State	N/A	12-month: 56.9	Low
Bagny et al.,^48^ 2013	Togo, western Africa	Lifetime prevalence of all types of exposure to BBF	155 nurses in Lome Campus Teaching Hospital	Lifetime: 34.8	ND	Moderate
Mashoto et al.,^49^ 2013	United Republic of Tanzania, eastern Africa	12-month prevalence of all types of exposure to BBF and disaggregated data on PCI	401 doctors, dentists, dental assistants, clinical officers, nurses, laboratory staff, radiologists, physiotherapists and health attendants in Tumbi and Dodoma regional hospitals	12-month: 47.9	12-month: 39.1	Low
Zoungrana et al.,^50^ 2014	Burkina Faso, western Africa	Lifetime prevalence of all types of exposure to BBF	275 student nurses and midwives in the medical ward of the Bobo-Dioulasso teaching hospital	Lifetime: 29.1	ND	Moderate
Beyera and Beyen,^51^ 2014	Ethiopia, eastern Africa	12-month prevalence of all types of exposure to BBF and disaggregated data on PCI	401 doctors, anaesthetists, nurses, laboratory staff, health officer and cleaners in four public health institutions (one hospital and three health centres) in Gondar city	12-month: 40.4	12-month: 22.9	Low
Yenesew and Fekadu,^52^ 2014	Ethiopia, eastern Africa	Lifetime and 12-month prevalence of all types of exposure to BBF and disaggregated data on PCI	317 nurses, health officers, health assistants, doctors, laboratory technicians and dentists in health-care facilities, Bahir Dar town	Lifetime: 76.0; 12-month: 65.9	Lifetime: 45.9; 12-month: 29.0	Low
Aynalem Tesfay and Dejenie Habtewold,^53^ 2014	Ethiopia, eastern Africa	12-month prevalence of all types of exposure to BBF and disaggregated data on PCI	211 doctors, nurses, midwives, health officers and laboratory technicians in two hospitals and two health centres in Debre Berhan town, Amhara region	12-month: 56.7	12-month: 31.5	Low
Beyene and Tadesse,^54^ 2014	Ethiopia, eastern Africa	12-month prevalence of all types of exposure to BBF	532 health-care workers in two hospitals and six health centres run by the government in Hawassa Town, southern Ethiopia	12-month: 51.9	ND	Low
Ajibola et al.,^55^ 2014	Nigeria, western Africa	Lifetime prevalence of PCI	300 doctors and nurses in Lagos University Teaching Hospital	N/A	Lifetime: 47.3	Moderate
Amira and Awobusuyi,^56^ 2014	Nigeria, western Africa	Lifetime and 12-month prevalence of PCI	102 doctors, nurses, dialysis technicians and auxiliary health staff in four (two government and two private) dialysis units in Lagos	N/A	Lifetime: 40.2; 12-month: 24.5	Moderate
Ogoina et al.,^57^ 2014	Nigeria, western Africa	Lifetime prevalence of all types of exposure to BBF	230 doctors, nurses and laboratory staff in two tertiary hospitals in north-central and south-south Nigeria	Lifetime: 84	ND	Moderate
Mbah,^58^ 2014	South Africa, southern Africa	12-month prevalence of all types of exposure to BBF	515 doctors and nurses in public, primary health-care settings in subdistrict F of Johannesburg metropolitan district	12-month: 25.2	ND	Low
Bekele et al.,^59^ 2015	Ethiopia, eastern Africa	Lifetime prevalence of PCI	340 doctors, anaesthetists, health officers, nurses, midwives, laboratory personnel, laundry workers and waste handlers in four hospitals in Bale zone, south-east Ethiopia	N/A	Lifetime: 37.1	Low
Burmen and Osoga,^60^ 2015	Kenya, eastern Africa	Lifetime prevalence of all types of exposure to BBF	116 laboratory staff	Lifetime: 77	ND	High
Arheiam and Ingafou,^61^ 2015	Libya, northern Africa	12-month prevalence of PCI	340 dental practitioners	N/A	12-month: 35.1	Low
Kone and Malle,^62^ 2015	Mali, western Africa	Lifetime prevalence of all types of exposure to BBF	128 doctors, nurses and students in a public hospital in Ségou, south-western Mali.	Lifetime: 64.1	ND	Moderate
Kateera et al.,^63^ 2015	Rwanda, eastern Africa	Lifetime prevalence of PCI	378 doctors, nurses and laboratory and support staff in the University Teaching Hospital of Butare, Huye District, Southern Province, Rwanda	N/A	Lifetime: 57.1	Moderate
Chalya et al.,^64^ 2015	United Republic of Tanzania, eastern Africa	12-month prevalence of all types of exposure to BBF and disaggregated data on PCI	436 doctors, nurses, laboratory staff and auxiliary health workers in Bugando Medical Centre, Mwanza	12-month: 48.6	12-month: 31.7	Low
Mponela et al.,^65^ 2015	United Republic of Tanzania, eastern Africa	12-month prevalence of all types of exposure to BBF and disaggregated data on PCI	291 doctors, dental staff, nurses, laboratory staff, medical attendants and cleaners in one referral and two district hospitals, Mbeya region	12-month: 35.1	12-month: 22.0	Low
Kassa et al.,^66^ 2016	Botswana, southern Africa	Lifetime prevalence of all types of exposure to BBF	1624 doctors, nurses and laboratory technicians in three public hospitals: a referral hospital and two district hospitals	Lifetime: 67.2	ND	Moderate
Kaweti and Abegaz,^67^ 2016	Ethiopia, eastern Africa	Lifetime and 12-month prevalence of PCI	496 doctors, nurses, laboratory technicians and cleaners in two public hospitals: Hawassa Referral and Adare District hospitals	N/A	Lifetime: 46; 12-month: 28	Low
Oluwatosin et al.,^68^ 2016	Nigeria, western Africa	Lifetime prevalence of PCI	642 doctors, nurses, laboratory workers and health attendants in two specialist hospitals in Ondo State	N/A	Lifetime: 55.8	Moderate
Nmadu et al.,^69^ 2016	Nigeria, western Africa	Lifetime prevalence of all types of exposure to BBF	172 nurses, midwives, community health workers and laboratory technicians in 14 primary health-care centres in Kaduna State	Lifetime: 68.9	ND	Low
Makhado and Davhana-Maselesele,^70^ 2016	South Africa, southern Africa	12-month prevalence of all types of exposure to BBF	233 nurses in a regional hospital in Limpopo Province	12-month: 43	ND	Low
Lahuerta et al.,^71^ 2016	United Republic of Tanzania, eastern Africa	Lifetime prevalence of all types of exposure to BBF and disaggregated data on PCI	973 doctors, nurses, dentists, students, cleaners and other support workers in three public hospitals	Lifetime: 79	Lifetime: 37	Low
Shindano et al.,^72^ 2017	Democratic Republic of the Congo, central Africa	12-month prevalence of all types of exposure to BBF	217 doctors and nurses in Bukavu, an eastern town in the Democratic Republic of the Congo	12-month: 42.8	ND	Low
Sharew et al.,^73^ 2017	Ethiopia, eastern Africa	12-month prevalence of PCI	195 nurses, midwives, laboratory staff, doctors, health officers and anaesthetists in two hospitals in Debre Berhan town, north-eastern Ethiopia	N/A	12-month: 32.8	Low
Laisser and Ng’Home,^74^ 2017	United Republic of Tanzania, eastern Africa	12-month prevalence of all types of exposure to BBF and disaggregated data on PCI	277 doctors, nurses and laboratory and auxiliary staff in 31 private and public health facilities in Kahama District, north-western United Republic of Tanzania	12-month: 59.2	12-month: 34.7	Moderate

AIDS: acquired immunodeficiency syndrome; BBF: blood and body fluid; N/A: not applicable; ND: not determined; PCI: percutaneous injury; STD: sexually transmitted disease.

^a^ All studies were cross-sectional.

^b^ The risk of bias was assessed as described in the methods.

**Fig. 2:**
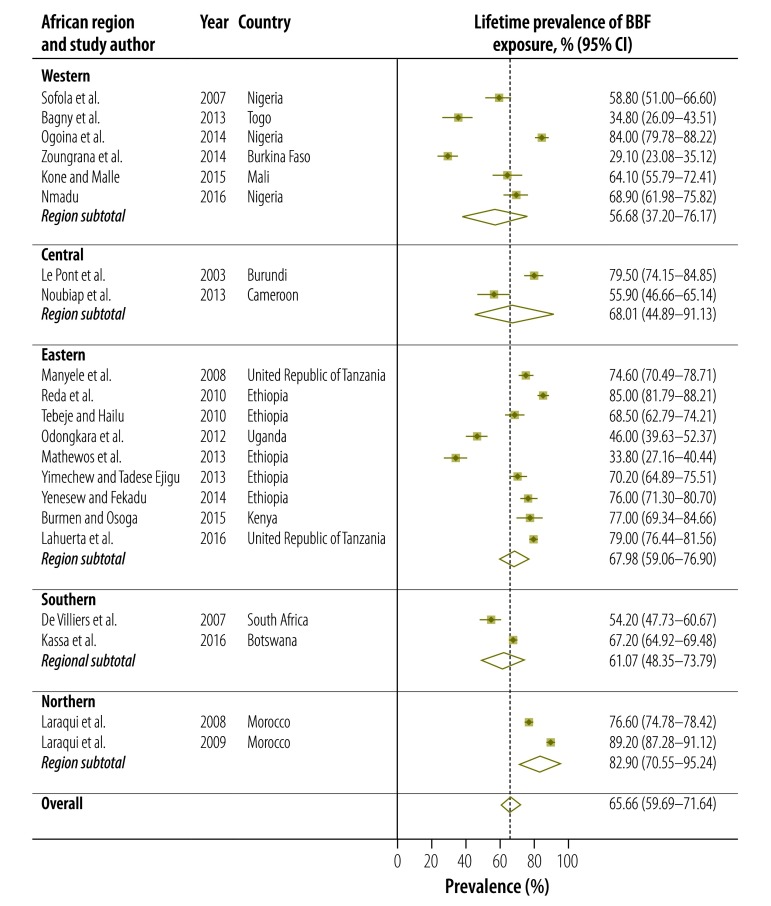
Meta-analysis, lifetime prevalence of blood and body fluid exposure among health-care workers in Africa, by region, 2002–2017

BBF: blood and body fluid; CI: confidence interval.

Note: The dashed vertical line presents the overall estimated prevalence.

**Fig. 3:**
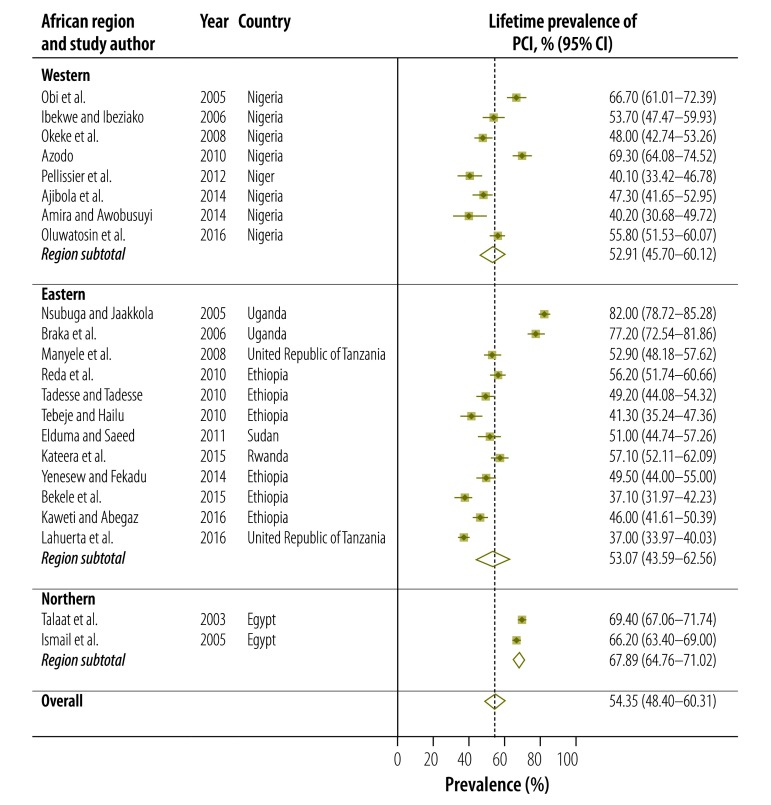
Meta-analysis, lifetime prevalence of percutaneous injury among health-care workers in Africa, by region, 2000–2017

CI: confidence interval; PCI: percutaneous injury.

Note: The dashed vertical line represents the overall estimated prevalence.

Twenty-one studies presented data on the lifetime prevalence of all types of occupational exposure to blood and body fluids, including percutaneous injury and mucous membrane exposure, among health-care workers in Africa (Table 1; available at: http://www.who.int/bulletin/volumes/95/12/17-195735). Lifetime prevalence varied widely from 29.1% (95% confidence interval, CI: 23.1–35.1) in Burkina Faso^50^ to 89.2% (95% CI: 87.3–91.1) in Morocco (Fig. 2).^29^ Overall, the estimated pooled lifetime prevalence was 65.7% (95% CI: 59.7–71.6). The regional prevalence estimate was highest for northern Africa: 82.9% (95% CI: 70.6–95.2). For percutaneous injury only, the lifetime prevalence ranged from 37.0% (95% CI: 34.0–40.0) in a Tanzanian study^71^ to 82.0% (95% CI: 78.7–85.3) in a Ugandan study (Fig. 3).^19^ Overall, the estimated pooled lifetime prevalence of percutaneous injury was 54.4% (95% CI: 48.4–60.3). After excluding studies with a high risk of bias, the estimated pooled lifetime prevalence of all types of exposure to blood and body fluids and of percutaneous injury was 65.1% (95% CI: 59.0–71.3) and 53.6% (95% CI: 47.3–60.0), respectively, figures which were comparable to the overall pooled estimates.

**Fig. 4:**
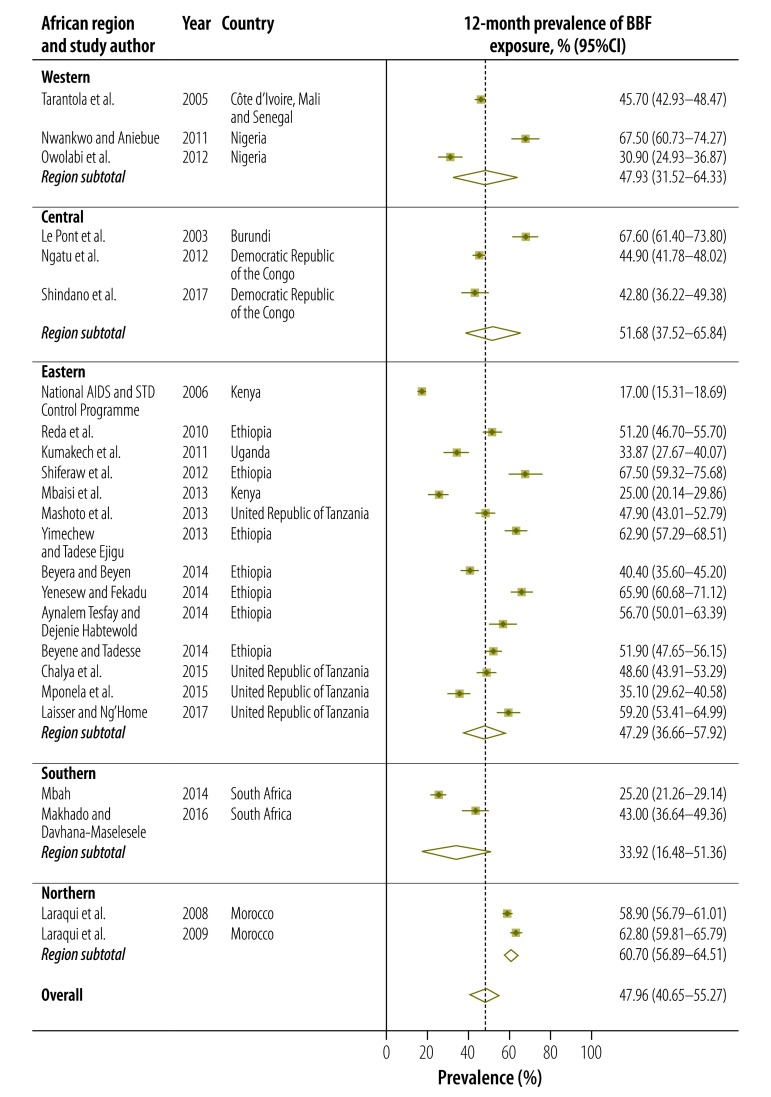
Meta-analysis, 12-month prevalence of blood and body fluid exposure among health-care workers in Africa, by region, 2002–2017

AIDS: acquired immunodeficiency syndrome; BBF: blood and body fluid; CI: confidence interval; STD: sexually transmitted disease.

Note: The dashed vertical line represents the overall estimated prevalence.

**Fig. 5:**
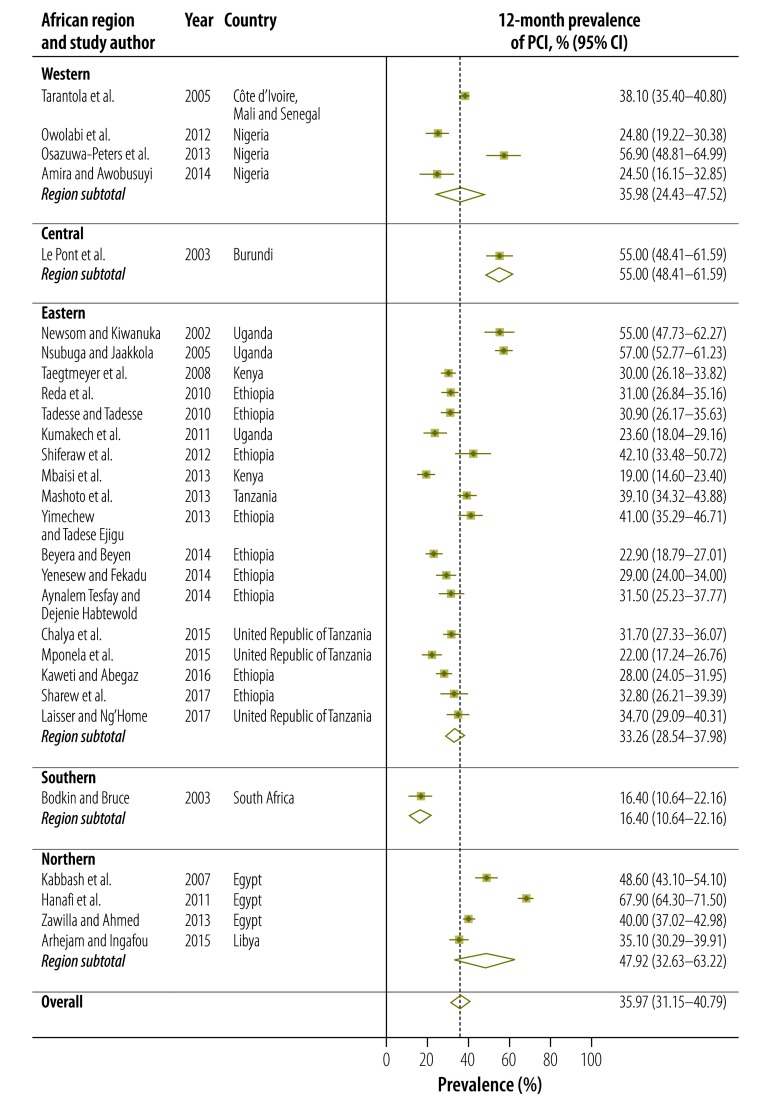
Meta-analysis, 12-month prevalence of percutaneous injury among health-care workers in Africa, by region, 2000–2017

CI: confidence interval; PCI: percutaneous injury.

Note: The dashed vertical line represents the overall estimated prevalence.

The 12-month prevalence of all types of occupational exposure to blood and body fluids ranged from 17.0% (95% CI: 15.3–18.7) in a Kenyan study^20^ to 67.6% (95% CI: 61.4–73.8) in a Burundian study (Fig. 4).^9^ The estimated pooled 12-month prevalence was 48.0% (95% CI: 40.7–55.3). Regional pooled estimates ranged from 33.9% (95% CI: 16.5–51.4) in southern Africa to 60.7% (95% CI: 56.9–64.5) in northern Africa. Twenty-eight studies reported the 12-month prevalence of percutaneous injury: it ranged from 16.4% (95% CI: 10.6–22.2) to 67.9% (95% CI: 64.3–71.5; Fig. 5). The pooled estimate was 36.0% (95% CI: 31.2–40.8). Seven studies provided disaggregated data on the 12-month prevalence of mucous membrane exposure: the pooled estimate was 18.2% (95% CI: 12.6–23.7).

**Fig. 6:**
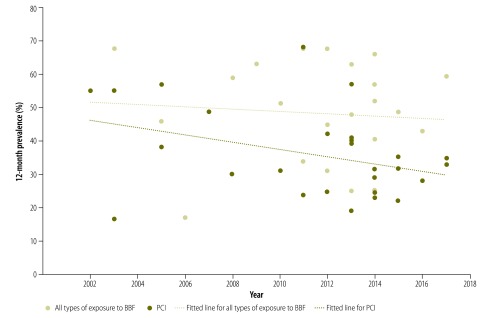
Trend in 12-month prevalence of blood and body fluid exposure and percutaneous injury among health-care workers in Africa, 2000–2017

BBF: blood and body fluid; PCI: percutaneous injury.

Note: The fitted lines were derived by linear regression.

In Fig. 6, the slopes of the fitted lines suggest that the 12-month prevalence of both all types of exposure to blood and body fluids and of percutaneous injury decreased only gradually over the study period. The estimated pooled 12-month prevalence for studies published between 2010 and 2017 was 47.3% (95% CI: 41.5–53.1) for all types of exposure and 33.7% (95% CI: 28.2–39.2) for percutaneous injury (Table 2). These estimates were comparable to the overall estimated pooled 12-month prevalence for all types of exposure and percutaneous injury, which were 48.0% (95% CI: 40.7–55.3) and 36.0% (95% CI: 31.2–40.8), respectively.

**Table 2:** Subgroup meta-analysis, blood and body fluid exposure and percutaneous injury among health-care workers in Africa, 2000–2017

Subgroup	Blood and body fluid exposure				Percutaneous injury		
	Pooled 12-month prevalence, % (95% CI)	No. studies included	Study heterogeneity, *I^2^,%* (*P*-value)		Pooled 12-month prevalence, % (95% CI)	No. studies included	Study heterogeneity, *I^2^,%* (*P*-value)
**African region**							
Western	47.9 (31.5–64.3)	3	96.8 (< 0.001)		36.0 (24.4–47.5)	4	94.1 (< 0.001)
Central	51.7 (37.5–65.8)	3	95.5 (< 0.001)		55.0 (48.4–61.6)	1	N/A
Eastern	47.3 (36.7–57.9)	14	98.7 (< 0.001)		33.3 (28.5–38.0)	18	94.0 (< 0.001)
Southern	33.9 (16.5–51.4)	2	95.4 (< 0.001)		16.4 (10.6–22.2)	1	N/A
Northern	60.7 (56.9–64.5)	2	77.0 (0.037)		47.9 (32.6–63.2)	4	98.3 (< 0.001)
**Study period**							
2000–2009	50.3 (29.2–71.4)	5	99.7 (< 0.001)		42.8 (32.7–52.8)	7	97.0 (< 0.001)
2010–2017	47.3 (41.5–53.1)	19	95.9 (< 0.001)		33.7 (28.2–39.2)	21	96.3 (< 0.001)
**Type of health-care facility**							
Hospital	49.7 (42.8–56.6)	14	97.0 (< 0.001)		39.2 (31.6–46.9)	17	97.6 (< 0.001)
Mixed^a^	47.8 (34.5–61.1)	9	99.1 (< 0.001)		31.5 (28.1–34.9)	9	81.7 (< 0.001)
**Risk of bias**							
Low	45.7 (36.7–54.6)	19	98.9 (< 0.001)		36.2 (30.5–41.8)	24	97.0 (< 0.001)
Moderate	56.4 (47.8–65.0)	5	94.5 (< 0.001)		35.2 (29.6–40.9)	4	73.3 (0.011)

BBF: blood and body fluid; CI: confidence interval; N/A: not applicable; PCI: percutaneous injury.

^a^ Both hospitals and primary care facilities.

Overall, substantial heterogeneity was observed among the studies for the estimated 12-month prevalence of all types of exposure to blood and body fluids (*X*^2^: 1816.5; *P* < 0.001; *I*^2^: 98.7%) and of percutaneous injury only (*X*^2^: 780.9; *P* < 0.001; *I*^2^: 96.5%). Meta-regression analysis showed that, of all the covariates explored in the bivariate analyses, only geographical region had a *P*-value less than 0.10: (P = 0.0874) and geographical region explained 17.6% of the between-study variation in the estimated 12-month prevalence of percutaneous injury.

### Subgroup analyses

As many of the studies included disaggregated data, we were able to estimate: (i) the pooled 12-month prevalence of occupational exposure to blood and body fluids by job category; and (ii) the relative risk of all types of exposure to blood and body fluids or of percutaneous injury between various demographic groups, which were distinguished, for example, by job category, gender, years of working experience or receipt of training on prevention of blood and body fluid exposure (details available from the corresponding author). The estimated pooled 12-month prevalence of exposure to blood and body fluids for medical doctors (excluding surgeons), nursing staff (including midwives and nursing assistants) and laboratory staff (including laboratory technicians) was 46.6% (95% CI: 33.5–59.7), 44.6% (95% CI: 34.1–55.0) and 34.3% (95% CI: 21.8–46.7), respectively. Moreover, when data on percutaneous injuries were included, there was no significant difference in the risk of all types of occupational exposure between these job categories: the relative risk (RR) was 1.108 (95% CI: 0.926–1.326) for doctors versus nursing staff, 1.267 (95% CI: 0.733–2.193) for doctors versus laboratory staff and 1.332 (95% CI: 0.947–1.874) for nursing staff versus laboratory staff. Nor was there a significant difference in risk between males and females (RR: 0.886; 95% CI: 0.692–1.133).

In addition, when data on percutaneous injuries were included, there was no significant difference in the risk of all types of occupational exposure between health-care workers with 5 years or less working experience and those with more than 5 years (RR: 0.999; 95% CI: 0.831–1.202). In contrast, health-care workers who worked 40 hours or more per week were significantly more likely to be exposed than those who worked fewer hours (RR: 2.221; 95% CI: 1.001–4.926). Six studies reported on health-care workers who had received training on infection prevention and occupational exposure to blood and body fluids. The risk of occupational exposure in the preceding 12 months among health-care workers without training was significantly higher than in trained staff (RR: 1.791, 95% CI: 1.234–2.071).

## Discussion

We found a high lifetime and 12-month prevalence of occupational exposure to blood and body fluids among health-care workers in Africa: about two thirds were exposed during their entire career and almost one half were exposed each year. Most exposure was due to percutaneous injury, which had an estimated 12-month prevalence of 36.0%. Direct comparison of our findings with those in other continents was difficult because of a lack of similar, continent-wide systematic reviews and meta-analyses. Nevertheless, the high prevalence of percutaneous injury among health-care workers in Africa has serious implications because most occupational exposure to blood-borne viruses, such as HBV and HIV, occurs via this route. This can have implications for the exposed health-care worker’s health, the transmission of blood-borne viruses to patients and the availability of scarce human resources for health care in Africa.

We found a variation in health-care workers’ exposure to blood and body fluids across Africa. Occupational exposure to blood and body fluids and percutaneous injury were consistently more frequent in northern Africa and less frequent in southern Africa. The reason for these regional differences is not clear. One possible explanation is that blood and body fluid exposure was underreported in some studies, which is likely. Alternatively, our findings may reflect regional differences in the level of knowledge of occupational exposure or in adherence to standard precautions.

Our meta-analysis found that the 12-month prevalence of blood and body fluid exposure differed little between various professions and there was no significant difference in risk. A critical appraisal of the literature showed that these figures may have been influenced by differences in study methods and in the categorization of health-care workers, but most discrepancies observed were linked to the underreporting of blood and body fluid exposure.^75^^,^^76^ In contrast, we found that the risk of blood and body fluid exposure was higher among health-care workers who had received no training on infection prevention, which is unsurprising because training improves knowledge and preventive practice. Furthermore, the risk of occupational exposure was also increased among staff who worked more than 40 hours per week. The acute shortage of health-care workers in Africa may, therefore, have contributed to the present findings.^4^ Inadequate staffing often results in a high patient-to-staff ratio, which may in turn lead to staff having to work longer hours to bridge gaps in personnel.^77^ Although longer hours can bring additional rewards for health-care workers, levels of stress and fatigue can increase, which may result in overworked staff becoming less alert and more susceptible to exposure to blood and body fluids.^77^ Our findings may therefore indicate the need not only to promote the safety and well-being of existing health-care workers in Africa, but also to address the acute shortage of health-care workers across the continent.

Our study also highlights the need to step-up efforts to reduce occupational exposure to blood and body fluids – particularly via percutaneous injury – among health-care workers in Africa. Percutaneous injury could be prevented by practical interventions such as safety engineered devices, including needleless intravenous systems, auto-disable syringes and blunt suture needles. However, our findings suggest that it may be more cost–effective to address factors contributing to increased exposure in the continent, such as a lack of training and long work hours. Regular in-service training for health-care workers could help promote standard precautions for preventing the transmission of blood-borne infection, such as hand hygiene, the use of personal protective equipment and techniques for minimizing the manipulation of sharps, including the avoidance of needle recapping. In addition to training health-care workers, a holistic strategy is needed to address the acute shortage of health-care workers in the continent and to monitor staff workload. Furthermore, standard precautions could be supplemented by educating health-care workers to take responsibility for their own health and safety and for that of others who may be affected by their actions at work. Finally, governments should provide policies and support systems for the surveillance, reporting and management of occupational exposure to blood and body fluids among health-care workers.

This study has some limitations. First, the cross-sectional design of the studies reviewed does not allow causal relationships to be established. Second, because the studies reviewed were based on self-reported retrospective data, they may be prone to recall and social desirability biases. Therefore, it is likely that exposure was underreported in many studies. Third, our review included single or limited reports from some countries and many reports concerned regional studies that were not nationally representative of the study countries. These factors may affect the generalizability of our findings. Furthermore, our review would have benefited from the inclusion of studies from Guinea, Liberia and Sierra Leone, where there was substantial transmission of Ebola virus infection among health-care workers during the recent outbreak. However, no studies of the prevalence of occupational exposure to blood and body fluids among health-care workers in these countries have been published. Future research in these countries should investigate occupational exposure to blood and body fluids and the circumstances in which it occurs to inform policy and practice. Nevertheless, our study provides an insight into the burden of occupational exposure to blood and body fluids among health-care workers in Africa and could prompt the development of appropriate policies, systems and processes in the continent.
